# Maternal health care use among married women in Hossaina, Ethiopia

**DOI:** 10.1186/s12913-015-1047-1

**Published:** 2015-09-10

**Authors:** Zeleke Dutamo, Nega Assefa, Gudina Egata

**Affiliations:** College of Medicine and Health Sciences, Semera University, Semera, Ethiopia; College of Health and Medical Sciences, Haramaya University, P.O.Box 1449, Dire Dawa, Ethiopia

## Abstract

**Background:**

Pregnancy and child birth are natural process of continuity of life. For many it is a normal process, for some it puts life at risk impending complications. Provision of skilled care for all women before, during, and after childbirth is a key in saving women’s life and ensuring delivery of healthy baby. Maternal health service drop-out through the course of pregnancy is widely claimed, yet by how much it is dropped is not known. The main aim of this study was to identify the use of maternal health service over the course of pregnancy and child birth in a comprehensive manner.

**Methods:**

A community based cross-sectional quantitative study on 623 women supported by qualitative inquiry was conducted Hossaian town, South Ethiopia during January 1–31, 2014. A structured questionnaire was used to generate the quantitative data and 4 Focus Group Discussions (FGD) were carried out to support the finding. Multiple logistic regression was used to control the effect of confounding. Odds ratios with 95 % CI used to display the result of analysis. Data generated from the FGD was analyzed using thematic analysis.

**Results:**

The study revealed that 87.6 % of women attended at least one antenatal care (ANC). Among 546 women who attended ANC, 61.3 % of the women made their first visit during second and third trimester of pregnancy and 49 % had less than four antenatal visits. The study also revealed that 62.6 % of deliveries were assisted by skilled attendants and 51.4 % of the women received at least one postnatal check-up. Parity, pregnancy intention and awareness on danger signs of pregnancy during pregnancy were significantly associated (*p* < 0.05) with ANC usage. Skilled delivery attendance was significantly associated with some socio-demographic, economic and obstetric factors. Average family monthly income, awareness on obstetric danger signs of pregnancy during recent pregnancy, and frequency of ANC were positive predictors of Postnatal Care (PNC) utilization.

**Conclusions:**

Though use of maternal health care services is relatively higher, however, it is not adequate. Engaging women in their own reproductive health affairs, strengthening maternal health care, increasing community awareness about obstetric danger signs during pregnancy and child birth, and telling the benefit of family planning should be major targets for intervention.

## Background

Maternal and Child Health care (MCH) is a primary focus of health care since the declaration of Alma-Ata in 1978. It includes health services during pregnancy, birth and postnatal period [1, 2]. Pregnancy and child birth are natural and continuous process in which some women are at risk for developing complication during pregnancy and child birth. In (2010), an estimated 287,000 women died around the world from largely preventable and treatable pregnancy and childbirth-related causes. Developing countries shares 99 % of the burden in which around 56 % of these maternal deaths took place in sub-Saharan Africa. According to the Ethiopian Demographic and Health Survey (EDHS) 2011, the maternal mortality ratio in Ethiopia was estimated to be 676 per 100,000 live births, which is much higher than many Sub-Sahara African countries [3–5].

The provision of skilled care before, during, and after childbirth saves life of women, and it also ensures the chance of having healthy infant [6, 7]. According to EDHS 2011 report, only 34 % of women received antenatal care from a skilled provider, for their most recent pregnancy [5]. The same report indicated that only 10 % of deliveries and 8.5 % of women given postnatal care by skilled professional. These figures are much lower for rural areas as compared to the urban. This implies that the utilization of maternal health care services in Ethiopia is low [5, 7, 8].

The main reason for such low coverage of skilled maternity care is associated with availability, accessibility, accommodation, affordability and acceptability of the service [9]. In Ethiopian context, through the health extension program, an intensive effort has been made to avail the service at the door steps of every person [10, 11]. However, in most instances it is not used to the fullest due to several reasons. The mix of available service, poor linkage between the service points, transportation, and sense of seriousness among health professionals compromises the health care.

So far, few studies showed a comprehensive analysis of maternal health care throughout the course of pregnancy, delivery and postnatal period [12–14]. Further, understanding in detail factors affecting maternal health care use is crucial. Therefore, this study tried to identify the level and determinants of maternal health care use among currently married women who gave birth in last 12 months in Hosanna town, South west Ethiopia.

Evidences from this study will help program planners and health executives at different level of service to design proper health care and implement interventions to the much affected segment of the population. As a result, it will increase the uptake of maternal health services and reduce suffering from problems arising during the course of pregnancy and child birth.

## Methods

### Study design and area

A cross-sectional quantitative study supported by qualitative inquiry was conducted among married women of reproductive age who gave birth during January 1 to December 30, 2013 in Hossana town. Hosanna town is situated 232 kms Southwest of Addis Ababa, the capital of Ethiopia. According to municipal report, in 2011, there were 84,433 people in the town and of these 49.2 % were females. The town has the three districts consisting in total eight Kebeles (smallest administrative unit in Ethiopia with a population of 5000 people). In the town, there is one hospital, three health centers, eight health posts and five private clinics. All governmental health facilities and three private clinics provide maternal health care services. These services include antenatal care, delivery care, and postnatal care.

### Sample size and sampling procedure

The quantitative survey was conducted on 634 married women who gave birth in the last 12 months prior to data collection. Data was collected during January 1–30, 2014. A multi-stage sampling technique was used to select study participants. First, from the three districts in town, two Kebles from the two big districts (Sech duna and Gofer meda) and one kebele from the smaller district (Addis kifle ketema) were selected using simple random sampling.

In the five selected Kebeles, census was conducted to identify households with eligible (women who had given birth in the last one year before data collection period). During the census it was learnt that the total households with eligible women registered were more than the sample size need, therefore, a proportional allocation for the five Kebeles was done to decide the number of women required from each Kebele.

Finally to identify women to be included in the survey, simple random sampling technique was applied. When more than one eligible respondent is present in selected household one respondent is selected at spot by a lottery method. For the qualitative data, purposive sampling was used to select two FGD pair (two among women and two among husbands) participants, in each FGD there were 8 participants.

### Data collection

Data was collected by twelve trained female data collectors who completed 12^th^ grade. The principal investigator trained intensively data collectors and supervisors for three days on the purpose and procedures of the survey. Quantitative data was collected using a structured interview questionnaire which is adapted from JHPIEGO [15]. The questionnaire was prepared in English and then translated to Amharic and local language (Hadiyisa). The interview questionnaire had three parts: socio-demographic and economic parts, women’s autonomy and questions related to the history of recent pregnancy and child birth. The questionnaire was pretested in one of the nearby Kebele. Field workers and investigators participated during pretest. Field supervisors and principal investigator closely supervised the data collection process. The qualitative data was generated using a semi-structured discussion guide prepared in English and translated into local language. The discussion was carried out using local language; it was tape recorded and note taken. On daily basis the discussions were analyzed to frame with the themes set from the objectives. Data generation, transcription, and analysis were carried out by experts with prior experience of handling qualitative data.

### Ethical consideration

The study obtained ethical approval from Institutional Research Ethics Committee (IREC) of College of Health and Medical Sciences, Haramaya University. Data collectors explained carefully the purpose of the study and the right of the respondents being included in the study. Participants were assured the confidentiality of their responses throughout the research process and thereafter. Informed written consent was secured before the start of data collection.

### Data analysis

Data was entered into Epi-Data version 3.02 software and exported to the SPSS version 20.0 for analysis. Descriptive analysis like percentages, proportions, mean and standard deviation were used to describe study population. We also conducted bivariate analysis to calculate the crudes odds ratio (OR) and with 95 % CI to determine association between dependent and independent variables. Furthermore, we performed multiple logistic regression analysis to control the effect of confounders. Variables with a *p* < 0.3 in the crude analysis were put in the final analysis. The dependent variables were antenatal, delivery and postnatal care whereas the independent variables are maternal age, women’s education, husband’s education, family income, women’s employment status, parity, pregnancy intention, and awareness of obstetric danger signs of pregnancy.

Antenatal care was recorded as received if the women had received antenatal care check-up at least once during the recent pregnancy. Delivery attendants are referred as skilled if the delivery was attended by Nurses, midwives, doctors and health officer. Similarly, PNC is defined as received if she had a postnatal check up for at least once from skilled health personnel. Thematic analysis was conducted for qualitative study to identify factors affecting the use of maternal health care.

## Result

A total of 634 currently married women who gave birth in the last one year prior to the date of data collection were interviewed and 623 questionnaires were responded properly that gives a response rate of 98.2 %. Majority (84.8 %) were in the age group of 20–34 the age distribution has mean and standard deviation of 27.8 and 5.3. More than half (58.3 %) of the respondents were protestant followed by Orthodox followers. Out of total respondents, 58 % of the women were Hadiya followed by Silte (13.6 %) and Kembata (13 %). Regarding women’s literacy, 41.3 % of women attended elementary education and 47.7 % of women attended secondary and above education whereas, 11 % of the respondents never attended formal school. More than half (57.9 %) of the respondents were unemployed while, 42.1 % were employed (Table 1).

**Table 1:** Socio-demographic and economic characteristics of respondents in Hosanna town, SNNPR, South West of Ethiopia January, 2014

Variables	Frequency (*n* = 623)	Percentage (%)
Age		
15–19	44	6.4
20–34	524	84.8
35–49	55	8.8
Mean ± SD		27.8 ± 5.26
Religion		
Protestant	363	58.3
Orthodox	131	21.0
Muslim	81	13.0
Catholic	39	6.3
Others^a^	9	1.4
Ethnicity		
Hadiya	358	57.5
Kembata	81	13.0
Silte	85	13.6
Amhara	85	9
Others^b^	43	6.9
Women’s education		
No education	69	11.1
Elementary school	257	41.3
Junior or high school	186	29.9
College/higher	111	17.8
Husband education		
No education	31	5.0
Elementary school	161	25.8
Junior or high school	242	38.8
College/higher	189	30.3
Women’s autonomy		
Higher	509	81.9
Lower	114	18.3
Employment status		
Unemployed	361	57.9
Employed for cash	218	35.0
Employed for non cash	44	7.1
Average family monthly income		
<450	101	16.2
450–110	242	38.8
>110	280	44.9
Mean ± SD		1,162.44 ± 669.70

^a^Atheist, Jehovans witness

^b^Gurage, Wolayita, Oromo, Tigre

### Maternal health care utilization

Out of 623 women, 87.6 % of the respondents had attended ANC at least one times during the last pregnancy. About half (51.7 %) of the women made their first antenatal visit in their second trimester, 26.3 % made during their first trimester, and the rests (9.6 %) of women attended in the third trimester of pregnancy. Of those who attended ANC at least once, majority asked about frequency of ANC attendance, and 49 % had less than four antenatal contacts and the rests, (38.7 %) reported to have four or more antenatal visits during last pregnancy.

**Fig. 1:**
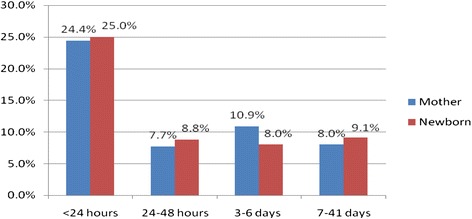
Distribution of timing of postnatal check up for the mother (*n* = 320) and the newborn (*n* = 318) in Hosanna town, SNNPR, South West of Ethiopia January, 2014

Regarding delivery attendance, more than half (62.6 %) of the deliveries were assisted by skilled health personnel whereas 37.4 % by non skilled person. The study also revealed that 64 % of deliveries took place in the health institutions while the remaining 34 % women delivered at home. About half (51.4 %) of the respondents received postnatal check up at least once from skilled health personnel, of which 24.1 % were examined within first 24 h of delivery, 7.7 % within 24–48 h, 10.9 % within 3–6 days, and 8.3 % within 7–41 days of delivery. In line with this, 51 % of newborns received postnatal checkup at least once after birth from skilled health professional. Out of babies who received the postnatal checkup, 25.2 % were examined within first 24 h after delivery, 8.8 % within 25–48 h, 8 % within 3–6 days and 9.1 % within 7–41 days (Fig. 1).

### Reasons for non attendance of antenatal and postnatal care

**Fig. 2:**
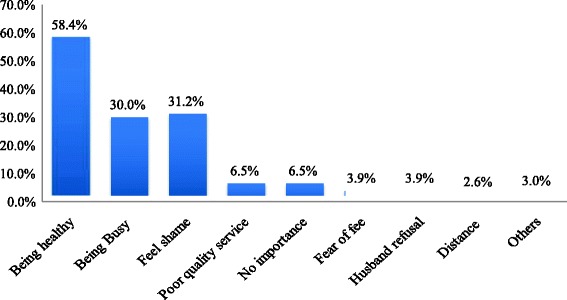
Reasons for non attendance of antenatal care visit in Hosanna town, SNNPR, South Western, South West Ethiopia January, 2014 (*n* = 77)

**Fig. 3:**
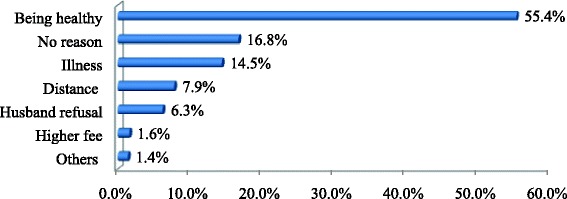
Reasons for non attendance of postnatal checkup in Hosanna town, South Western Ethiopia January, 2014 (*n* = 303)

Out of 77 non users of antenatal care, more than half (58.4 %) reported that being healthy is the major reason for not following up ANC. Other reasons for not attending ANC were work overload, feeling shame to attend ANC, poor quality of the service, and not knowing the importance ANC (Fig. 2).the major reasons for not attending postnatal care were no health problem to attend, illness, and cost to obtain the services (Fig. 3).

In the FGDs, the majority of participants identified being in a state of healthy condition, work overload, not knowing the importance of ANC, cost and trust on local TBAs were the major reasons for not attending ANC. Similarity apparently healthy, no reason for attendance, illness, cost of services, husband refusal, distance from the health facility were the main reasons for non attendance of postnatal care. Majority discussants did not feel it is necessary to attend ANC and PNC care as most of the time they did not experience any problems during pregnancy and postnatal period.

*A 27 year female discussants said, “I did not go to the health facilities anymore (during pregnancy) because I felt healthy”. “I have two children before and I didn’t go to any health institution to attend ANC because nothing happened during my prior pregnancy and delivery." *

*Another a 24 women discussant said, “I feel healthy. Nothing happened to me. You need money to go health institution. If I am not sick why should I go to checkup (after delivery)? I do not want to waste my money and time”.*

### Factors associated with maternal health care use

Parity, pregnancy intention and awareness of danger signs of pregnancy were associated with ANC use. The odd of using ANC was 2. 4 (2.4, 95 % CI 1.1, 5.2) times among women having one child compared to women having five and more children and women having 2–4 children were 2.8 times more likely to use ANC (AOR 2.8, 95 % CI 1.4,5.6). The odd of ANC use among women whose last pregnancy was intended was 2 times (AOR 2.0, 95 % CI 1.1, 3.6) than that unintended pregnancy. Women who were aware of at least one danger signs of pregnancy during recent pregnancy were seven times more likely to attend ANC than that of the reference group (not aware) (AOR 7.0, 95 % CI 3.8, 13.0) (Table 2).

**Table 2:** Factors associated with maternal health care services utilization among mothers in Hosanna town during 2013, SNNPR, 2014

Explanatory variables					
			Adjusted odds ratio (95 % CI)		
		n	ANC	DC	PNC
Age group of women	15–19	44	1.1 (.3, 4.1)	1.5 (.468, 4.9)	.5 (.1, 1.2)
	20–34	524	1.4 (.6, 3.3)	1.15 (.5, 2.5)	.9 (.4, 2.0)
	35–49	55	1.0	1.0	1.0
Mother’s education	No education	69	1.0	1.0	1.0
	Elementary	257	1.49 (.6, 3.4)	.4 (.2, .9) *	.5 (.3,1.2)
	Secondary	186	1.80 (.7, 4.7)	.3 (.1, .8) *	.6 (.3,1.2)
	College/higher	111	2.1 (.5, 8.5)	.3 (.1, .7) **	.6 (.2, 1.5)
Husband education	No education	31	1.0	1.0	1.0
	Elementary	161	.83 (.3, 2.3)	2.5 (.9, 7.5)	.6 (.2, 1.7)
	Secondary	242	1.77 (.6,5.0)	1.4 (.6, 4.8)	.4 (.1, 1.2)
	College/higher	189	1.4 (.4, 4.4)	4.4 (1.4, 13.6)**	.7 (.2, 2.0)
Employment status	Unemployed	361	1.0	1.0	1.0
	Employed for cash	218	1.7 (.8,3.3)	.8 (.5,1.3)	1.3 (.7,2.7)
	Employed for non cash	44	2.7 (.6,12.5)	.7 (.3, 1.4)	1.0 (.5, 2.2)
Women’s autonomy	Higher	509	1.6 (.8, 2.9)	1.4 (.8, 2.4)	.9 (.5, 1.6)
	Lower	114	1.0	1.0	1.0
Average family monthly income	<450	101	.7 (.3, 1.7)	.6 (.3, 1.1)	.6 (.3, 1.2)
	450–110	242	1.1 (.5, 2.3)	.7 (.4, 1.1)	.6 (.4, .9) *
	>1100	280	1.0	1.0	1.0
Parity	1	207	2.4 (1.1,5.2)**	.9 (.4, 2.0)	1.0 (.5,1.962)
	2–4	328	2.8 (1.4,5.6)*	.8 (.4, 1.7)	.9 (.5, 1.7)
	5^+^	88	1.0	1.0	1.0
Pregnancy intention	Intended	504	1.96 (1.1,3.6)*	1.8 (1.1, 3.2)*	1.5 (.9, 2.7)
	Unintended	119	1.0	1.0	1.0
Aware danger signs of pregnancy	Yes	407	7.0 (3.8,13.0)***	3.9 (2.6, 6.0)***	3.6 (2.4, 5.5)***
	No	216	1.0	1.0	1.0
ANC	Yes	546		1.7 (.6, 5.2)	1.2 (.4, 3.5)
	No	77		1.0	1.0
Frequency of ANC	≤3	305		1.0	1.0
	≥4	241		1.6 (1.1, 2.5)*	1.6 (1.1, 2.4)**

*ANC*: Antenatal care, *DC*: Delivery care, *PNC*: Postnatal care

*** = p < 0.001, ** = p < 0.01,* = p < 0.05)

Women’s educational level of college/higher, educational status of husband, pregnancy intention, awareness to pregnancy danger signs and frequency of antenatal care were significantly associated with skilled delivery attendance. Women’s literacy status was positively associated with the skilled delivery care utilization. Women who were not educated were less likely to be assisted by skilled health worker during delivery than women who were elementary, secondary and college/higher educated. The odds of utilizing delivery care among women whose husband education status was college/higher were 4.4 times compared to their counterparts (AOR 4.4, 95 % CI 1.4, 13.6) (Table 2).

The odds of skilled delivery care utilization among women with intended pregnancy were about two times than that of their counterparts. Women who were aware at least one danger signs of pregnancy were 4.4 times more likely to use skilled delivery care. Women who attended ANC at least four times were 1.6 times more likely to be assisted by skilled health worker during delivery compared to women who attended ANC at most three times (AOR 1.6, 95 % CI 1.1, 2.5) (Table 2).

Average family monthly income of 500–1100 Birr, awareness on pregnancy danger signs and frequency of antenatal were significantly associated with postnatal cares use. Women whose average family monthly income was between 500 and 110 were less likely to receive postnatal checkup compared to their counterparts. Women who were aware of at least one danger sign of pregnancy were 3.6 times more likely to utilize PNC (AOR 3.6, 95 % CI 2.4, 5.5). Women who attended ANC at least four times were more likely to receive PNC from skilled health workers after delivery than women who attended ANC at most three times (AOR 1.6, 95 % CI 1.1, 2.4) (Table 2).

## Discussion

The study revealed that antenatal, delivery and postnatal care utilization were 87.6 %, 62.6 % and 51.4 %, respectively. Availability in terms of mix and quality; geographical accessibility; accommodation of specific women needs; affordability of cost of service and other costs related to it; and cultural and religious acceptability of maternal health service affects the uptake of health care by the women [9]. In Ethiopian context, through the health extension program, intensive effort has been made to reach everyone including pregnant and delivering women [10, 11]. It has basic goals of health promotion and disease prevention [16, 17]. However, in most instances it is not used to the fullest due to several reasons. The mix and quality of available service, consistency of service, poor linkage between the service points, poor access for transport, and lack of sense of urgency among health professionals compromises the health care.

In this study, parity, pregnancy intention and awareness of obstetric danger signs were significantly associated with ANC utilization. Women’s educational level, pregnancy intention, awareness of danger signs of pregnancy, and frequency of ANC visit were significantly associted with skilled delivery care utilization. Whereas, average family monthly income of 450–1100 Birr, awareness on obstetric danger signs of pregnancy, and frequency of ANC visit for the recent pregnancy are significantly associated with skilled postnatal care use. Readers are advised to take into consideration, to some extent miss reporting of age as there are no reliable sources of such information in Ethiopia. And secondly recall bias might have affected some of the reporting.

Out of the total women included in the survey, who have delivery reporting in 2013, 87.6 % of women attended at least one ANC from skilled health care providers. The finding is in line with study conducted in Holeta town (87.1 %) and Woldia (89 %) [12, 18], and slightly higher than the study conducted in North Gondar zone (32.3 %) and Sidama zone (77.4 %) [14, 19]. This might be the fact that studies were conducted in both rural and urban kebeles where the distance from health institution could be a major predictor of ANC utilization in rural areas. This study finding is also slightly higher than the EDHS (2011) that showed 76 % women living in urban areas received professionally assisted ANC [6]. This might be due to the fact that in our study area there is an increase in the awareness among women due to recently started urban health extension program which is providing equitable access to promotive, preventive and selective curative health interventions through health extension program [10, 11]. It is also important to note the time gap between the EDHS 2011 and the current study.

About 63 % of the women were assisted by skilled health professional during delivery. This finding is higher than the study conducted in Holeta town (61.6 %) but, slightly higher than the study conducted in Woldia (48.3 %) [12, 18]. This might be due to the difference in the nature of the study site. The study done in Woldia included women from both rural and urban part of the study area but current study was conducted in urban only which indirectly indicating the distance between home and the health facilities might be a contributing factor. It is also, slightly higher compared to the DHS (2011) in Ethiopia showed that women living in urban received professionally assisted delivery care was about 51 % [5]. This could be the fact that awareness creation among women about the benefit of skilled attendance at delivery through expansion of urban health extension program and currently started free ambulance services in the community.

In this study about 51.6 % of the women received at least one postnatal care from skilled health personnel. This study result is higher compared to the study conducted in North Gondar (6.3 %) [13]. The difference could be in North Gondar study, women included in the study were from both urban and rural parts, but ours were only urban residents. It is also higher than the DHS (2011) report of Ethiopia which is 32.1 % urban women received postnatal checkup at least once from skilled health worker. This is might be due to an increase in awareness of women by urban extension health program and an increase in ANC. There is an increased chance of women who had ANC to use postnatal checkup [20, 21]. Women who had fewer children are more likely to attend ANC compared to the reference group (women having 5 and above children). This result is consistent with the previous studies conudcted in Ethiopia and other developing countries [13, 18, 22]. Low utilization of ANC among high parity women could be because multiple responsibilities to care children, limited resources in the family and negative perceptions resulting from previous pregnancies and child births.

This study finding also revealed the odds of utilizing ANC and DC among women whose pregnancy intended are higher compared to the reference group. The positive effect of intended pregnancy on maternal health care utilization was observed in different studies done elsewhere [22–24]. This might be due to the fact that women with unintended pregnancies may initially attempt to deny their pregnancies to themselves. As the result they are less motivated to seek ANC and DC services. Recognition of obstetric danger signs of pregnancy by women and their family could prevent significant maternal morbidity and mortality and promptly seek health care [2, 3]. Women who were aware at least one obstetric danger sign of pregnancy are more likely to utilize ANC, DC and PNC compared to the women who didn’t. Similar effect was observed in study conducted in Ethiopia and other developing countries [25, 26].

In this study, the primary reason given for not attending ANC services include being in a state of good health, being too busy, no or little knowledge about ANC, poor quality of services, husband refusal and far distance from home to health service and etc. Other studies also reported similar reasons [18, 22]. The main reasons for non-attendance of ANC given by most FGDs participants were being in a state of health, work overload, not knowing the importance of ANC, cost to get the services, and trust on local TBAs.

Maternal education has positive effect on skilled delivery attendance. This finding is consistent with other studies [12, 18, 27]. Skilled birth attendance was significantly associated with the level of husband education. That is, women who had college/higher educated husbands are more likely to use safe delivery services than those with no education levels. Other studies are consistent with these findings [12, 27, 28]. This survey also revealed that women who attended ANC at least four times were more likely to seek skilled delivery than women who attended at most three times. Similar findings were reported in Woldia and Southern Tanzania [12, 29]. This may be due to the fact that women with more ANC visits showed a higher satisfaction with the care quality and hence more likely to use health services for delivery. It is also fact that women with many ANC visits were exposed for more health education and counseling which increases the service utilization.

Concerning postnatal care, women whose average family monthly incomes 450–1100 Birr were less likely to receive postnatal checkup compared to women whose monthly income was above 1100 Birr. A high family monthly income increases the opportunity to contact with others and get information about modern health care services including maternal health care services. Other studies indicated that women who attended ANC at least four times during pregnancy were more likely to receive postnatal checkup compared to women who had received ANC 1–3 times. This finding is in line with the study conducted in Uttarakhand and in Kenya [21, 30]. The increment in ANC visits will expose the women to increased health education and counseling on the importance and the benefit of postnatal check from skilled health professional which are both likely to increase service utilization of postnatal care.

## Conclusion

Majority of the women had attended at least one ANC visit from skilled health care providers during their recent pregnancy however; less than half of the women received the recommended four antenatal visits by WHO. More than half of the deliveries were attended by skilled health professional and about half of the women received postnatal checkup at least once within 41 days of delivery. Parity, pregnancy intenion and awareness on danger signs of pregnancy were factors associated with ANC. Women’s and husband’s literacy status, pregnancy intention, awareness on danger signs of pregnancy and frequency of ANC attendance were predictors of DC utilization while average family monthly income, awareness on danger signs of pregnancy and the frequency of ANC were factors associated with PNC utilization.

Improving the status of women by expanding educational opportunities, strengthening promotion of maternal health care about the importance and benefits through mass media and community health education, strengthening of community awareness program with the focus on obstetric danger signs of pregnancy, family planning and child spacing were important in improving maternal health care utilization.
